# Assessing the Potential Distribution of *Pseudoechthistatus* (Coleoptera: Cerambycidae) in China Under Climate Change Using Species Distribution Models

**DOI:** 10.1002/ece3.71303

**Published:** 2025-04-14

**Authors:** Liang Zhang, Ping Wang, Guang‐Lin Xie, Wen‐Kai Wang

**Affiliations:** ^1^ Institute of Entomology, College of Agriculture Yangtze University Jingzhou People's Republic of China; ^2^ MARA Key Laboratory of Sustainable Crop Production in the Middle Reaches of the Yangtze River (Co‐Construction by Ministry and Province), College of Agriculture Yangtze University Jingzhou People's Republic of China

**Keywords:** biodiversity conservation, climate change, habitat suitability, MaxEnt, *Pseudoechthistatus*

## Abstract

Climate change will lead to changes in biological ecosystems, which may affect the geographic distribution of *Pseudoechthistatus* and thus alter the extent and spatial pattern of its habitat. *Pseudoechthistatus* plays an important role in biodiversity and has significant ecological value. This study utilized an optimized MaxEnt model to predict the predicted distribution of *Pseudoechthistatus* in China for the current and future (2050s and 2070s). The results show that the MaxEnt model has high prediction accuracy with AUC values higher than 0.97 for both training and testing. The most influential factors contributing to the distribution of *Pseudoechthistatus* were temperature seasonality (Bio4) and isothermality (Bio3), accounting for 38.8% and 28.2%, respectively. Furthermore, southern China remains a region of high suitability for *Pseudoechthistatus* species diversity. However, the Beijing climate center climate system model (BCC‐CSM2‐MR) predicts a decrease in suitable areas for *Pseudoechthistatus*, while the model for interdisciplinary research on climate (MIROC6) predicts an increase in medium and low suitable areas for *Pseudoechthistatus*. Additionally, future climate change will significantly alter its distribution pattern, with *Pseudoechthistatus* predicted to decrease its suitable area by 6.64%–28.01% under the BCC‐CSM2‐MR model and increase its suitable area by 6.14%–18.61% under the MIROC6 model. The results show that the MaxEnt model can improve the understanding of the geographical distribution of *Pseudoechthistatus* in the context of climate change and provide a scientific basis for the identification of potentially suitable habitats and the development of stable suitable areas for conservation.

## Graphical abstract

Unfavorable climatic conditions have always threatened the distribution patterns of organisms. Therefore, in the context of global warming, the question of how insects will evolve in response to climate change has become important, especially for those species that are less abundant. To this end, we used an optimized maxent model to predict the potential distribution areas and how Pseudoechthistatus would migrate in China in the current and two future periods.

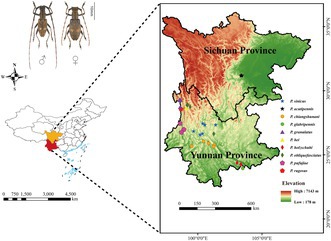

## 1 Introduction

The greenhouse effect, triggered by the global economy and human activities, is projected to result in a sustained increase in global temperatures in the future (Urban 2015). This warming trend is expected to trigger rising sea levels, the melting of Arctic ice, alterations in flora and fauna, and a heightened frequency of extreme weather events, particularly heat waves (Pound et al. 2021). The geographical distribution of species is an important spatial characteristic of species, which is the result of long‐term interactions between species and the environment (Sun et al. 2020). Preserving biodiversity and ensuring the stability of ecosystems are imperative for maintaining ecological equilibrium on a global scale (Zhang et al. 2023). Global climate change is profoundly affecting biodiversity, and the geographical distribution of many species has already changed significantly (Sun et al. 2020). According to the Intergovernmental Panel on Climate Change (IPCC), about 18% of insects could lose half of their climate‐suitable habitat with a 2°C rise in temperature (Harold et al. 2020). Insects are important components of ecosystems, playing key roles in material cycling, plant pollination, and food chain maintenance (Dawson et al. 2011). However, climate change is altering insect habitats, affecting their population dynamics, distributional range, and ecological functions (Urban 2015). Insects are also susceptible to the impacts of climate change, which can impede their migratory capabilities and potentially lead to population extinctions, as previous studies have shown (Duffy et al. 2022). Unfavorable climatic conditions have resulted in a reduction in the number of new species taxa and confined their ranges to smaller areas (Hardy et al. 2010). All assertions presented are based on verifiable scientific evidence. It has been shown that global warming has already led to a decline in forest insect diversity, with some similarities to the species extinction event at the end of the Permian. Therefore, in the context of a warming climate, there is an urgent need to understand how species distribution patterns will evolve in response to climate change in order to implement necessary conservation measures now, particularly for those rare insect groups that are less abundant.

China encompasses a vast geographical area and diverse topography, the southern region is considered one of the richest in biodiversity worldwide (Li et al. 2022). Especially, the area near the Yunnan‐Guizhou Plateau is a hotspot of biodiversity and biological evolution, and its alpine environment provides conditions for studying the high‐elevation adaptability of many species (Wang et al. 2022). The longhorn beetles are closely linked to their host plant, and this co‐evolution can serve as a bio‐indicator of forest health and species diversity (Hayat 2022; Hayat, Akram, et al. 2023; Zhang et al. 2024a). Moreover, beetles play an important role in nutrient cycling, maintaining habitat structure, and as indicator species (Luo et al. 2024). Therefore, longhorn beetles play an important role in the natural ecosystem and have significant economic and ecological value (Schneider et al. 2018). The beetle genus *Pseudoechthistatus* Pic 1917 (Coleoptera: Cerambycidae) has 11 species worldwide, 10 of which occur in China (Bi and Lin 2016; Wang, Xie, et al. 2019; Huang et al. 2020). Species of the genus *Pseudoechthistatus* are widely distributed in the second and third terrace regions of China, inhabiting fir broad‐leaved forests or mixed coniferous forests at altitudes of 1800–3000 m (Wang, Xie, et al. 2019). Adults of the *Pseudoechthistatus* tend to parasitize dead leaves and branches, feeding on dead leaves or bark of the fir and maple families. Furthermore, most species are nocturnal and hide in or around host plants during the day (Bi and Lin 2016). However, despite the potential ecological role of this genus in forest ecosystems, studies on its biological information are relatively scarce, which may stem from the rarity of new species of this genus on the one hand, and the insufficient knowledge of its suitable habitat and potential distribution range on the other (Wang, Xie, et al. 2019; Zhang et al. 2022). In addition, the southwest region has been identified as a potentially suitable area for the discovery of new populations or species of the genus (Wang et al. 2022). Therefore, the application of ecological niche modeling to explore the habitat suitability, distribution patterns, and potential migration pathways of insects of the genus *Pseudoechthistatus* under different climate scenarios in the future is crucial for revealing their environmental adaptations. This will not only help to fill the gap in the ecological study of this taxon but also provide a scientific basis for biodiversity conservation and ecological management.

Understanding the relationships between species and their natural environments is fundamental to unraveling the ecological requirements and spatial distribution patterns of organisms (Denan et al. 2020). The distribution of species is influenced by a combination of factors, including bioclimatic conditions, forest dynamics, and human activities, which determine habitat suitability and the long‐term viability of populations. Insects are particularly sensitive to environmental change, with temperature and precipitation being key factors influencing their ecological adaptation and geographical distribution, which together shape their habitat patterns through direct and indirect effects (Lux et al. 2022). Temperature not only directly regulates the metabolism, developmental rate, and reproductive capacity of insects, but also indirectly shapes their ecological adaptive strategies by altering the growth of host plants (Zhang, Cai, et al. 2021). Appropriate temperatures may accelerate the life cycle of insects and improve reproductive efficiency, but too high temperatures may increase the physiological stress of host plants and weaken the resource base on which insects depend for survival, thereby affecting their population dynamics. Similarly, precipitation and its seasonal variations affect insect survival and dispersal capacity by regulating environmental humidity and plant growth rhythms (Zhao et al. 2023). Higher precipitation may provide more stable habitats, whereas reduced precipitation or extreme drought may inhibit vegetation growth, thereby limiting the extent of insect habitats. However, in addition to temperature and precipitation, altitudinal gradients have equally profound effects on the geographical distribution and ecological niche adaptation of insects. Higher elevations may limit the migratory capacity of species and affect their population dynamics and survival strategies. In addition, human activities, such as deforestation, urban sprawl, and agricultural development, can significantly alter the habitat characteristics of insects, which in turn affect their range (Hill et al. 2017). Together, these factors interact to shape insect habitat patterns and their responses to environmental change.

With the continuous development of computers and geographic information systems (GIS), species distribution models (SDMs) have been continuously improved and refined, and many researchers have used ecological niche models (ENMs) in the prediction of potential geographical distribution of species (Iannella et al. 2021; Zhang et al. 2024b). Among the many SDMs, the MaxEnt model has robust simulation and prediction capabilities and is thus widely used in various types of research (Wang, Xu, et al. 2023; Zhang et al. 2024c). The principle of the MaxEnt model is to calculate the optimal distribution of a species under the constraints of a certain ecological niche, that is, the possible distribution of the species in a certain prediction area when entropy is maximized, based on the principle of climatic similarity and the corresponding environmental variables of the species (Harte et al. 2021; Soliman et al. 2023). Examples include predicting potential distributions of species of different taxa (e.g., mammals, birds, amphibians, and insects), center‐of‐mass migration, and determining fitness intervals for environmental variables using response curves (Lee et al. 2021; Meza Mori et al. 2023; Zhang et al. 2024d). Therefore, the MaxEnt model is widely used in the prediction of potential distribution areas of rare and endangered species with little distribution data and narrow distribution ranges (Zhang et al. 2023). In this study, we use the MaxEnt model to simulate the potential suitability areas of *Pseudoechthistatus*.

In this study, we applied the optimized MaxEnt model with two climate models (BCC‐CSM2‐MR and MIROC6) to predict the current and future (2050s and 2070s) suitable distribution areas of the genus *Pseudoechthistatus* in China. The study objectives include: 1. To evaluate the potential distribution pattern of the genus *Pseudoechthistatus* under current and future climate scenarios. 2. To identify bioclimatic factors affecting the genus *Pseudoechthistatus*. 3. To explore the similarities between two future climate models and the current period. 4. To analyze the migration direction and dispersal pattern of the genus *Pseudoechthistatus* in the future. The results of this study will enable us to understand how future climate change will drive changes in the distribution pattern of the genus *Pseudoechthistatus* and provide scientific support for the development of conservation priority areas and the implementation of habitat management measures.

## 2 Materials and Methods

### 2.1 Data

#### 2.1.1 Species Distribution Data

**FIGURE 1:**
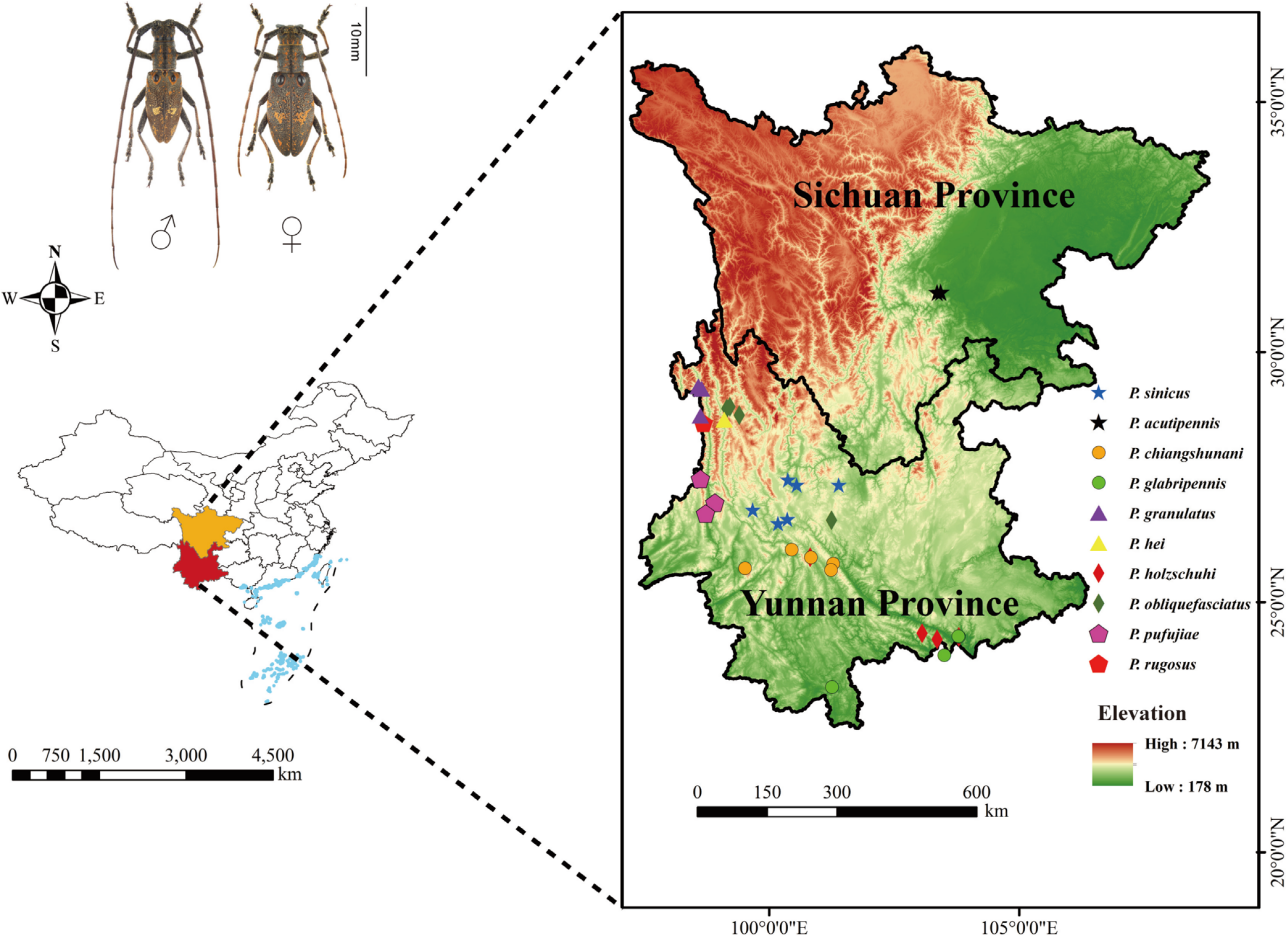
Occurrence records of *Pseudoechthistatus* in China.

To generate occurrence records of *Pseudoechthistatus* in China for modeling, data were obtained from published references (e.g., Bi and Lin 2016; Huang et al. 2020), online databases such as the Global Biodiversity Information Facility (GBIF, https://www.gbif.org/, accessed November 12, 2023) and iNaturalist (https://www.inaturalist.org/, accessed November 12, 2023), as well as field surveys conducted by our research team (Wang, Xie, et al. 2019). In addition, for sites lacking specific latitude and longitude coordinates, Online Google Earth (http://ditu.google.cn/) was used to obtain this information. Finally, we collected a total of 33 distribution records from multiple sources (Figure 1; Table S1).

Based on the objective fact that ecological niches between sister species are generally conserved over evolutionary time, we analyzed the *Pseudoechthistatus* at the genus level due to its limited availability. Although species‐level analyses could provide finer‐scale ecological insights, genus‐level analysis remains a valid approach for understanding broader biogeographic patterns, especially when species within the genus share similar ecological preferences and habitat requirements. This approach allows us to infer potential distributional trends while acknowledging the limitations of low species richness (Lwin et al. 2021). We used the “ENMTools” package (version 1.0.4) of the R (version 4.4.1) platform (https://www.r‐project.org/, accessed on October 1, 2023) to reduce over‐fitting or incorrect predictions resulting from spatial clustering of species records (Warren et al. 2021). The “ENMTools” package can automatically match the size of the environmental factor grid used for analysis and can delete redundant data within the same grid; we used 2.5 arc min (~5 km). This method is fast and efficient, and the analysis results are more reasonable. Consequently, the occurrence points obtained were necessarily smaller than the actual distribution area. Finally, 32 valid occurrence records were retained for the construction of the MaxEnt model. Nonetheless, the MaxEnt model selected in this work still has the characteristics of excellent prediction and accuracy in the case of a small sample size (Lee et al. 2021; Soliman et al. 2023).

#### 2.1.2 Environmental Variables

We downloaded 19 bioclimatic variables (Table 1) at a resolution of 2.5 arc minutes from the WorldClim global climate database (http://www.world‐clim.org, accessed on November 24, 2023) (Fick and Hijmans 2017; Wu et al. 2019). Current climate conditions are based on the 1970–2000 observational record, while future climate data are based on the Beijing climate center climate system model (BCC‐CSM2‐MR) and the model for interdisciplinary research on climate (MIROC6) system models developed in the Coupled Model Intercomparison Project 6 (CMIP6). The BCC‐CSM2‐MR climate model has a higher atmospheric and land surface resolution and better simulation of topographic precipitation and local temperature distributions, while MIROC6 has a finer atmospheric vertical resolution and incorporates shallow convective parameterisation (Guo et al. 2022). In addition, in terms of internal climate variability, MIROC6 simulates more realistically the interannual variability of the intraseasonally eastward‐propagating Madden‐Julian Oscillation (MJO) and interannual variations of the summertime East Asian monsoon are more realistically simulated in MIROC6. We selected four shared socio‐economic pathway (SSP) scenarios, namely, low forcing scenario (SSP1‐2.6), medium forcing scenario (SSP2‐4.5), medium‐high forcing scenario (SSP3‐7.0), and high forcing scenario (SSP5‐8.5), for future climate simulations in the 2050s and 2070s. These scenarios depict different socio‐economic development pathways based on actual development and planning in each country (Kaky et al. 2020).

**TABLE 1:** Correlation analysis and screening of bioclimatic variables.

Abbreviation	Environmental variables	|*r*| > 0.9	Operation
Bio1	Annual mean temperature (°C)	Bio6, Bio11	Eliminate
Bio2	Mean diurnal range (°C)	—	Retain
Bio3	Isothermality	—	Retain
Bio4	Temperature seasonality	Bio7	Retain
Bio5	Maximum temp of warmest month (°C)	Bio8, Bio10	Eliminate
Bio6	Minimum temp of coldest month (°C)	Bio1, Bio9, Bio11	Retain
Bio7	Temperature annual range (°C)	Bio4	Eliminate
Bio8	Mean temp of wettest quarter (°C)	Bio5, Bio10	Eliminate
Bio9	Mean temp of driest quarter (°C)	Bio6, Bio11	Eliminate
Bio10	Mean temp of warmest quarter (°C)	Bio5, Bio8	Retain
Bio11	Mean temp of coldest quarter (°C)	Bio1, Bio6, Bio9	Eliminate
Bio12	Annual precipitation (mm)	Bio13, Bio16, Bio18	Eliminate
Bio13	Precipitation of wettest month (mm)	Bio12, Bio16, Bio18	Retain
Bio14	Precipitation of driest month (mm)	Bio17, Bio19	Eliminate
Bio15	Precipitation seasonality (mm)	—	Retain
Bio16	Precipitation of wettest quarter (mm)	Bio12, Bio13, Bio18	Eliminate
Bio17	Precipitation of driest quarter (mm)	Bio14, Bio19	Retain
Bio18	Precipitation of warmest quarter (mm)	Bio12, Bio13, Bio16	Eliminate
Bio19	Precipitation of coldest quarter (mm)	Bio14, Bio17	Eliminate

The selection and treatment of bioclimatic factors is crucial when constructing species distribution models, as correlations between variables can lead to autocorrelation and multicollinearity problems, which in turn affect the predictive accuracy of the model. To address these issues, we first assessed the percentage contribution of 19 bioclimatic variables using the Jackknife method in the MaxEnt model (Figure S1). Then, Pearson correlation analyses were performed on the 19 bioclimatic variables using the “ENMTools” package in R software. Variables with a correlation coefficient of |*r*| ≤ 0.9 were selected for modeling to avoid the problem of multicollinearity. When the correlation coefficient of two variables was |*r*| > 0.9, we retained only the variables with higher percentage contribution to avoid the multicollinearity problem. After screening, the mean diurnal range (Bio2), isothermality (Bio3), temperature seasonality (Bio4), minimum temp of coldest month (Bio6), mean temp of warmest quarter (Bio10), precipitation of wettest month (Bio13), precipitation seasonality (Bio15), and precipitation of driest quarter (Bio17) were finally retained for model construction (Table 1).

### 2.2 Optimization of Model Parameters

In the model optimization process, “Feature Classes” (FCs) and “Regularization Multiplier” (RM) are the most important parameters in the MaxEnt model, and optimizing these two parameters can significantly improve the accuracy of the model outputs (Lee et al. 2021; Zheng et al. 2023). We optimized the MaxEnt (version 3.4.4) software (https: //biodiversityinformatics.amnh.org/open_source/maxent/, accessed on June 4, 2023) using the “Kuenm” package (version 1.1.10) (https://github.com/marlonecobos/kuenm, accessed on November 25, 2023) from the R software (Phillips et al. 2006). Different combinations of FCs and incremental RM values were set in the MaxEnt model calibration. The FCs contained five basic parameters: linear‐L, quadratic‐Q, product‐P, threshold‐T, and hinge‐H. During the calibration of the MaxEnt model, we set different combinations of FC and incremental RM values, taking into account the studies of Harte et al. (2021) and Soliman et al. (2023). We set a total of 12 regularization multipliers (0–6 with 0.5 intervals) and 6 feature combinations (L, LQ, H, LQH, LQHP, and LQHPT). To determine the final optimization parameters, we first screened the candidate models to retain those that were statistically significant. Second, the model set is refined based on the omission rate criterion. Finally, the optimal MaxEnt model with the smallest delta. AICc is selected from the 72 models (Figure S2) (Sun et al. 2020; Xian et al. 2023).

### 2.3 Species Distribution Model

Optimized MaxEnt was utilized to model species distributions and predict them for current and future (Yang et al. 2022). Due to data availability constraints, we referenced the approach based on Lwin et al. (2021) and Niknaddaf et al. (2023) with minor modifications. The final model was trained using 75% of the geographically distributed data and tested with 25% of the data, with the maximum number of iterations set to 10,000 for 10 repetitions, and cross‐validation was used to reduce the risk of model overfitting in order to improve the model's generalization ability (Zhang, Kass, et al. 2021). The area under the curve (AUC) of the receiver operating characteristic curve (ROC) is a core measure of model accuracy and predictive performance. The ROC curve is an acceptance curve with the horizontal coordinate indicating the false positive rate and the vertical coordinate indicating the true positive rate. The accuracy of the optimized MaxEnt model in predicting the *Pseudoechthistatus* distribution was assessed by AUC values. The AUC value ranges from 0 to 1, with larger values indicating higher predictive performance. Specifically, an AUC value below 0.6 indicates that the model results are not credible, between 0.6 and 0.7 indicates that the model performance is poor, between 0.7 and 0.8 is average, between 0.8 and 0.9 indicates that the model performance is good, and more than 0.9 implies that the model performance is very good (Harte et al. 2021; Aidoo et al. 2022; Yoon and Lee 2023). In addition, we used the Jackknife method of the MaxEnt model to calculate the contribution of each bioclimatic factor to assess the influence of environmental variables on the suitability predictions of *Pseudoechthistatus*.

### 2.4 Change of Suitable Area Under Different Climates

The results of the MaxEnt model were output in logistic format and then imported into ArcGIS Map software (version 10.8.1) for visualization and analysis (Vale et al. 2021). First, the distribution values were divided according to the division method of assessing probability in the IPCC report (Yang et al. 2022). Then, habitat suitability was divided into four classes, which were represented by different colors: unsuitable area (*p* < 0.05, White), low suitability area (0.05 < *p* < 0.33, Green), medium suitability area (0.33 < *p* < 0.66, Yellow) and high suitability area (0.66 < *p* < 1, Red) (Gao, Qian, et al. 2023; Hayat et al. 2024). To further analyze the spatial migration patterns of the genus *Pseudoechthistatus*, we used the “Centroid Changes (Lines)” tool in the “SDMToolbox” (version 2.6) package to quantify its geocentric movement trajectories over time. This method is able to visualize the spatial and temporal dynamics of the species' suitable distribution areas, which supports the understanding of their potential migration trends (Aidoo et al. 2022; Li et al. 2022; Gao, Qian, et al. 2023).

### 2.5 Analysis of Multivariate Environmental Similarity Surface

The multivariate environmental similarity surface (MESS) was used to analyze the extent to which climate change and its main driving variables affect the potential suitability areas of *Pseudoechthistatus* in China. The environmental variables of *Pseudoechthistatus* contemporary potential fitness areas were used as reference layers, and the similarity between them was calculated for current and future climates. The similarity value (S) reflects the degree of similarity between the climatic conditions of a point and those of the reference layer during a specific period of time (Zhao et al. 2020; Niknaddaf et al. 2023). Negative values indicate that at least one of the values of the environmental variables at the point is out of the range of the corresponding value of the reference layer, which is called a climatic anomaly, while the maximum value of 100 means that the climate at the point is completely normal (Xian et al. 2023). This operation is realized by running the “density.tools.Novel” tool in the “maxent.jar” file in the command window, and the ASCII file exported from the model was imported into ArcGis Map (version 10.8.1) software for drawing.

## 3 Results

### 3.1 Reliability Analysis of Models Established and Influence of Bioclimatic Variables

The average AUC value of the optimized MaxEnt model (LQ = 1) was higher than that of the default parameter model (LQHP = 1) (Figure S2). Specifically, the average training AUC value of the optimized model is 0.975 and the average test AUC value is 0.976, both of which are close to 1.0, which suggests that the optimized MaxEnt model shows higher predictive ability and accuracy in predicting and testing the distribution of *Pseudoechthistatus* (Table 2). Moreover, we further checked the OR_10_ and found that its value is less than 0.1, which indicates that the model is well fitted and no obvious overfitting problem occurs.

**TABLE 2:** Model accuracy and performance evaluation.

Run	Training AUC	Testing AUC
Rep 1	0.975	0.999
Rep 2	0.971	0.968
Rep 3	0.972	0.985
Rep 4	0.983	0.949
Rep 5	0.971	0.966
Rep 6	0.983	0.964
Rep 7	0.973	0.992
Rep 8	0.972	0.970
Rep 9	0.978	0.980
Rep 10	0.972	0.983
Ave	0.975	0.976

*Note:* Run, number of runs.

Abbreviation: AUC, area under the curve.

In addition, by applying the Jackknife test and MaxEnt modeling, we calculated the percentage contribution and permutation importance of each environmental variable to assess their relative importance in the model. The results indicated that the most important bioclimatic variable was temperature seasonality (38.8%), followed by isothermality (28.20%), precipitation of the driest quarter (8.90%), and minimum temperature of the coldest month (5.10%). The cumulative contribution of these four variables was 81.00%, indicating that they were the main bioclimatic factors determining suitable habitat for *Pseudoechthistatus* (Table 3).

**TABLE 3:** The percentage contribution and permutation importance of environmental variables for predicting the distribution of *Pseudoechthistatus*.

Variable	Percent contribution	Permutation importance
Bio4	38.8	63.9
Bio3	28.2	7.3
Bio17	8.9	4.2
Bio6	5.1	13.8
Bio13	1.5	0.3
Bio15	1.1	0
Bio10	0.3	0
Bio2	0.2	0.1

### 3.2 Potentially Suitable Distribution Areas of *Pseudoechthistatus* Under the Current Climate

**FIGURE 2:**
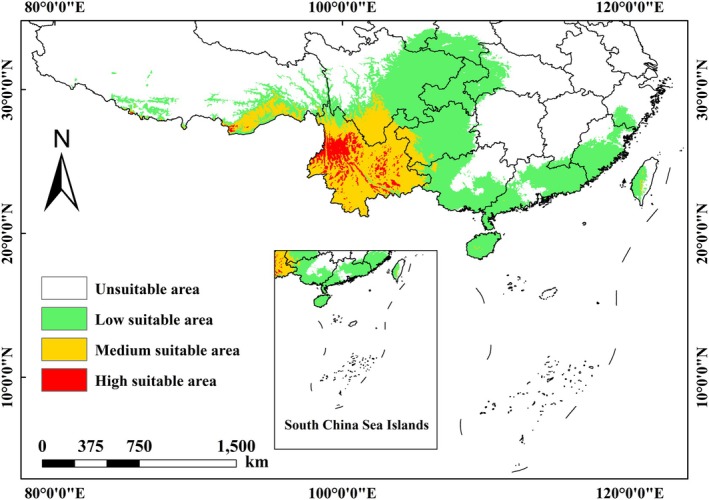
Habitat suitability of *Pseudoechthistatus* in China under current climate scenarios.

Under the current climatic conditions, the potential suitable areas for *Pseudoechthistatus* in China are mainly concentrated in the Yunnan‐Guizhou Plateau, the Himalayas, and the Lingnan region (Figure 2), with the areas of unsuitable and suitable areas being 809.93 × 10^4^ km^2^ and 50.62 × 10^4^ km^2^, respectively, which account for 84.21% and 15.79% of the total area (Table 4). The low suitability, medium suitability, and high suitability areas are 107.54 × 10^4^ km^2^, 37.57 × 10^4^ km^2^, and 6.73 × 10^4^ km^2^, respectively, representing 70.83%, 24.74%, and 4.43% of the suitable area (Table 4).

**TABLE 4:** Predicted suitable areas for *Pseudoechthistatus* under current and future climatic conditions.

General circulation model	Decade	Scenarios	Predicted area (10^4^ km^2^)			Comparison with current distribution (%)		
			Low suitable	Medium suitable	High suitable	Low suitable	Medium suitable	High suitable
Now	Current	—	107.54	37.57	6.73	—	—	—
BCC‐CSM2‐MR	2050s	SSP1‐2.6	87.62	34.73	3.04	−18.52	−7.56	−54.81
		SSP2‐4.5	105.52	34.71	1.53	−1.89	−7.61	−77.24
		SSP3‐7.0	74.79	33.17	1.85	−30.46	−11.71	−72.49
		SSP5‐8.5	81.02	29.65	0.60	−24.67	−21.08	−91.07
	2070s	SSP1‐2.6	74.02	34.05	1.25	−31.18	−9.38	−81.39
		SSP2‐4.5	81.09	30.85	1.19	−24.60	−17.89	−82.25
		SSP3‐7.0	88.29	31.19	1.71	−17.90	−16.99	−74.61
		SSP5‐8.5	82.32	29.43	1.66	−23.45	−21.66	−75.30
MIROC6	2050s	SSP1‐2.6	118.36	39.09	4.59	10.06	4.04	−31.79
		SSP2‐4.5	126.06	38.67	4.70	17.22	2.92	−30.17
		SSP3‐7.0	133.17	39.20	4.08	23.83	4.33	−39.33
		SSP5‐8.5	123.43	38.95	4.02	14.77	3.66	−40.21
	2070s	SSP1‐2.6	121.52	39.21	5.17	13.00	4.35	−23.20
		SSP2‐4.5	117.65	39.38	4.14	9.40	4.81	−38.48
		SSP3‐7.0	135.29	40.42	4.39	25.80	7.58	−34.71
		SSP5‐8.5	126.76	40.22	4.05	17.87	7.06	−39.85

### 3.3 Changes in Potentially Suitable Distribution Areas of *Pseudoechthistatus* Under the Future Climate Scenarios

**FIGURE 3:**
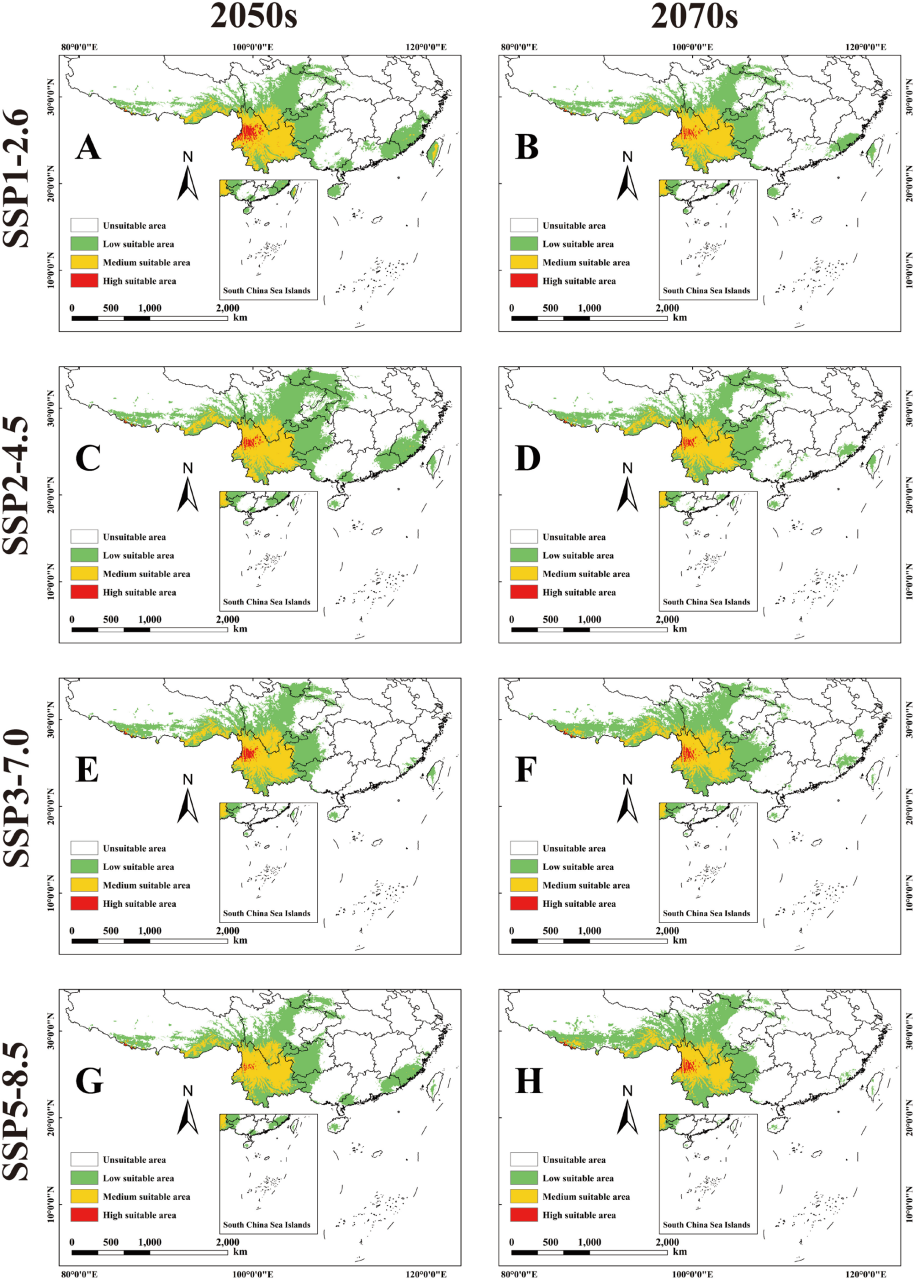
Habitat suitability of *Pseudoechthistatus* in China under future BCC‐CSM2‐MR climate model scenarios. Note: (A) SSP1‐2.6‐2050s; (B) SSP1‐2.6‐2070s; (C) SSP2‐4.5‐2050s; (D) SSP2‐4.5‐2070s; (E) SSP3‐7.0‐2050s; (F) SSP3‐7.0‐2070s; (G) SSP5‐8.5‐2050s; (H) SSPSSP5‐8.5‐2070s.

**FIGURE 4:**
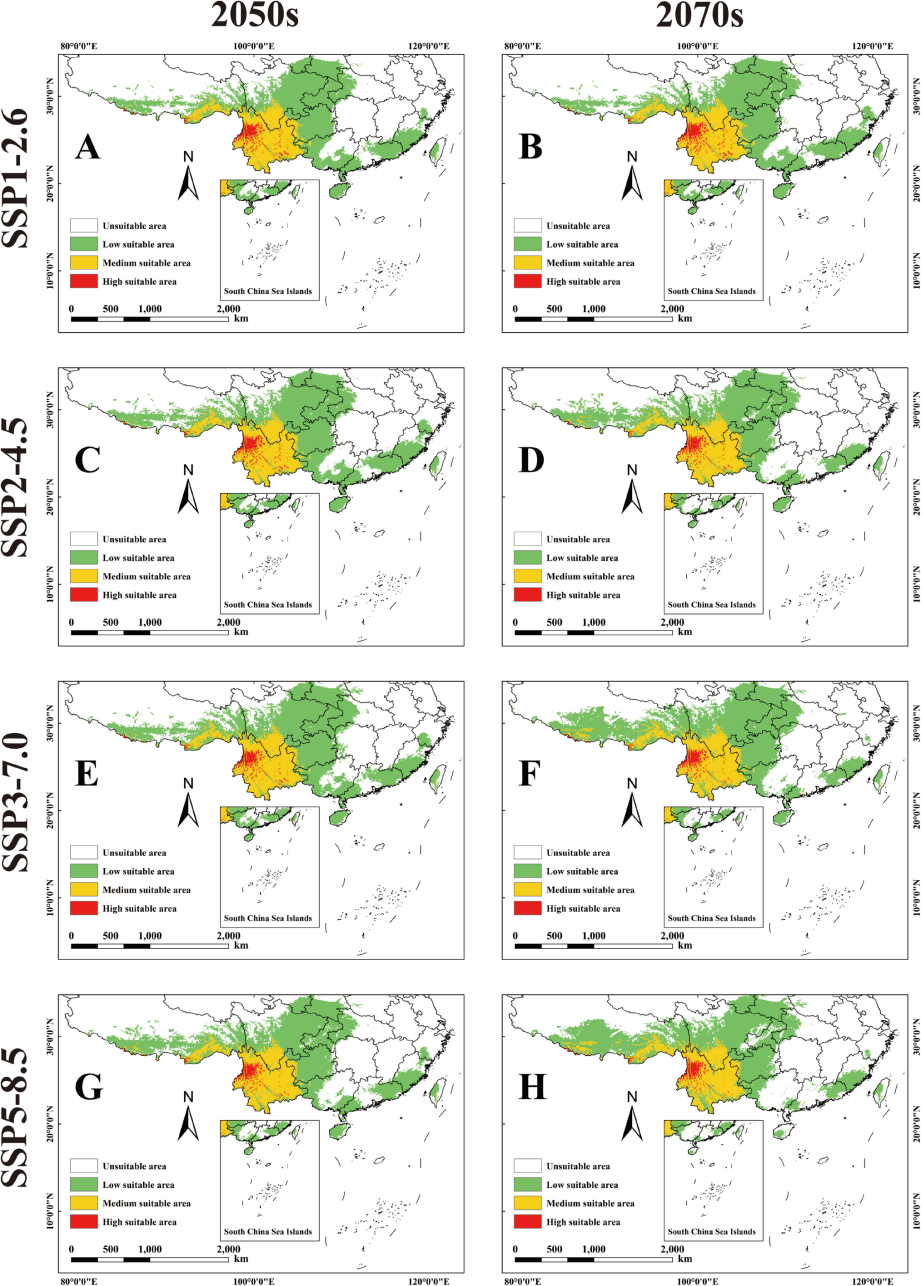
Habitat suitability of *Pseudoechthistatus* in China under future MIROC6 climate model scenarios. Note: (A) SSP1‐2.6‐2050s; (B) SSP1‐2.6‐2070s; (C) SSP2‐4.5‐2050s; (D) SSP2‐4.5‐2070s; (E) SSP3‐7.0‐2050s; (F) SSP3‐7.0‐2070s; (G) SSP5‐8.5‐2050s; (H) SSPSSP5‐8.5‐2070s.

Under the future BCC‐CSM2‐MR climate model, the potential habitat of the genus *Pseudoechthistatus* shows a significant contraction trend compared to the current one. Specifically, its suitable area ranged from 109.31 to 141.76 × 10^4^ km^2^, with the high suitable area shrinking from 54.81% to 91.07%, the medium suitable area shrinking from 7.56% to 21.66%, and the low suitable area shrinking from 1.89% to 31.18% (Figure 3; Table 4). In contrast, under the MIROC6 climate model, potential habitat for the genus *Pseudoechthistatus* increased significantly from the current. The size of suitable areas ranged from 161.16 × 10^4^ km^2^ to 180.10 × 10^4^ km^2^. While there was a general decrease in the size of the high suitability area, there was an increasing trend in the size of the medium and low suitability areas. Specifically, the contraction of high suitability areas ranged from 23.20% to 91.07%, while the area of medium suitability areas increased from 2.92% to 7.58%, and that of low suitability areas increased from 9.40% to 25.80% (Figure 4; Table 4).

### 3.4 Spatial Transfer Characteristics of Suitable Areas for *Pseudoechthistatus*

**FIGURE 5:**
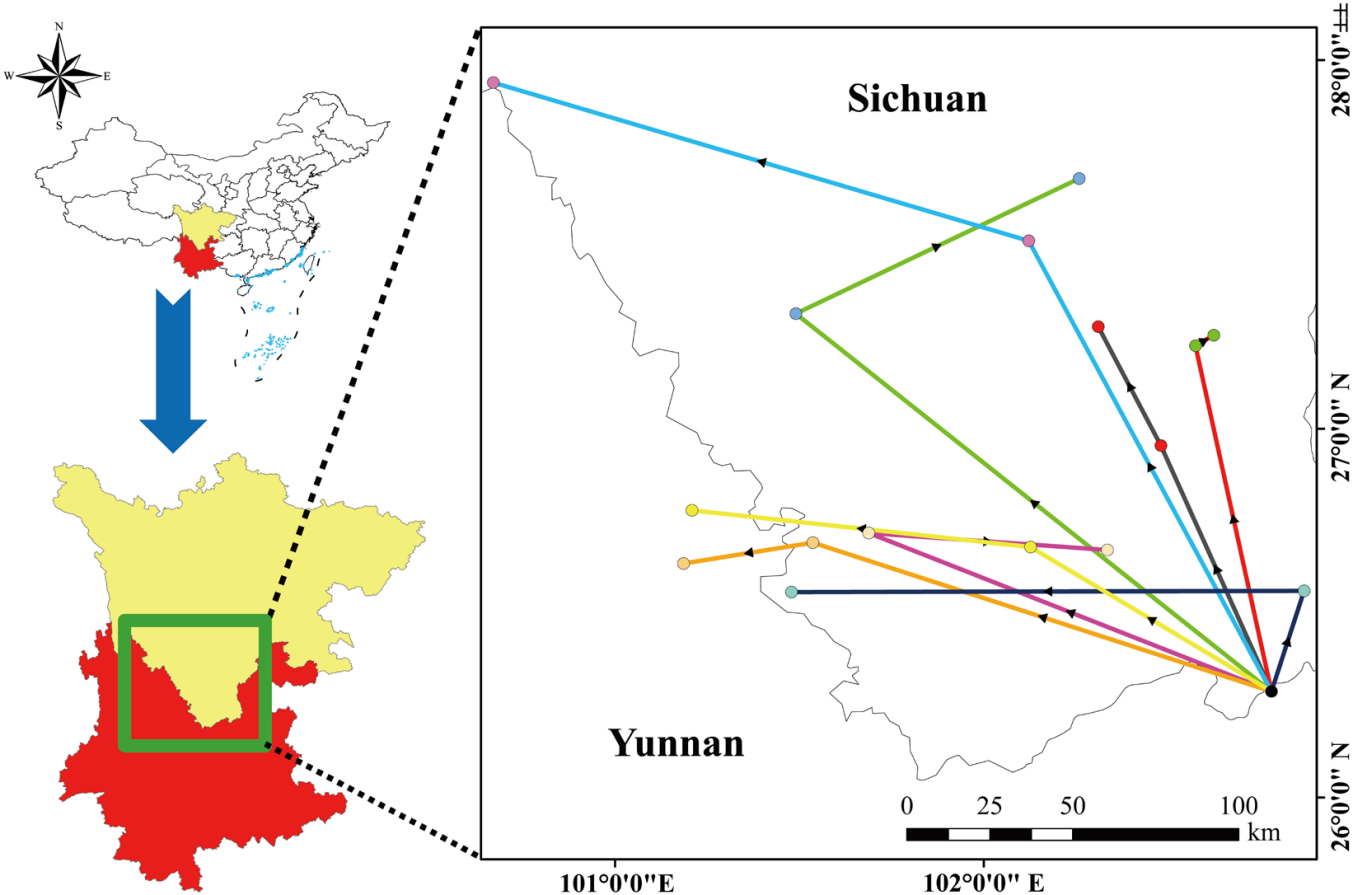
Potential distributional spatial movement paths of *Pseudoechthistatus* in China under different climate scenarios in the future. Note: The gray line represents the migration route of the center of distribution of *Pseudoechthistatus* in the BCC‐CSM2‐MR SSP1‐2.6 scenario; the red line represents the migration route of the center of distribution of *Pseudoechthistatus* in the BCC‐CSM2‐MR SSP2‐4.5 scenario; the green line represents the migration route of the center of distribution of *Pseudoechthistatus* in the BCC‐CSM2‐MR SSP3‐7.0 scenario; the light blue line represents the migration route of the center of distribution of *Pseudoechthistatus* in the BCC‐CSM2‐MR SSP5‐8.5 scenario; the dark pink line represents the migration route of the center of distribution of *Pseudoechthistatus* in the MIROC SSP1‐2.6 scenario; the orange line represents the migration route of the center of distribution *Pseudoechthistatus* in the MIROC SSP2‐4.5 scenario; the dark blue line represents the migration route of the center of distribution of *Pseudoechthistatus* in the MIROC SSP3‐7.0 scenario; the yellow line represents the migration route of the center of distribution of *Pseudoechthistatus* in the MIROC SSP5‐8.5 scenario.

Under future climate change scenarios, the potential habitat centers of *Pseudoechthistatus* show different migration trends. In the current period, the distribution center of *Pseudoechthistatus* is located in Yunnan Province (26.28° E 102.78° N). With the influence of climate change, the distribution center of *Pseudoechthistatus* is expected to migrate to higher latitudes and altitudes under future climate models and is expected to gradually expand to areas such as Sichuan Province, especially under the SSP5‐8.5‐2070s scenarios of the two climate models, which showed the farthest migration distance (Figure 5). Specifically, under the BCC‐CSM2‐MR climate model, the migration distance of *Pseudoechthistatus* range centers ranged from 89.07 km to 307.60 km. Under the MIROC6 climate model, the migration distance of *Pseudoechthistatus* distribution centers ranged from 34.54 km to 184.47 km, which indicates that the response of species suitability areas varied under different climate models (Table 5).

**TABLE 5:** Alternational trends of longitude, latitude, and migration distance of *Pseudoechthistatus* in different periods.

General circulation model	Decade	Shared socioeconomic pathways	Longitude	Latitude	Center migration distance (km)
Now	Current	—	102.78	26.29	—
BCC‐CSM2‐MR	2050s	SSP1‐2.6	102.48	26.95	89.07
		SSP2‐4.5	102.31	27.28	117.97
		SSP3‐7.0	102.57	27.23	189.66
		SSP5‐8.5	102.62	27.25	167.24
	2070s	SSP1‐2.6	101.49	27.31	133.26
		SSP2‐4.5	102.26	27.68	121.71
		SSP3‐7.0	102.12	27.51	181.19
		SSP5‐8.5	100.67	27.94	307.60
MIROC6	2050s	SSP1‐2.6	101.69	26.72	132.67
		SSP2‐4.5	102.33	26.67	145.53
		SSP3‐7.0	101.54	26.69	34.54
		SSP5‐8.5	101.19	26.63	86.45
	2070s	SSP1‐2.6	102.87	26.56	68.27
		SSP2‐4.5	101.48	26.56	181.71
		SSP3‐7.0	102.13	26.68	147.81
		SSP5‐8.5	101.21	26.78	184.47

### 3.5 Multivariate Environmental Similarity Surface (MESS) of 8 Bioclimatic Variables Affecting *Pseudoechthistatus* Distribution Under Future Climate Scenarios

**FIGURE 6:**
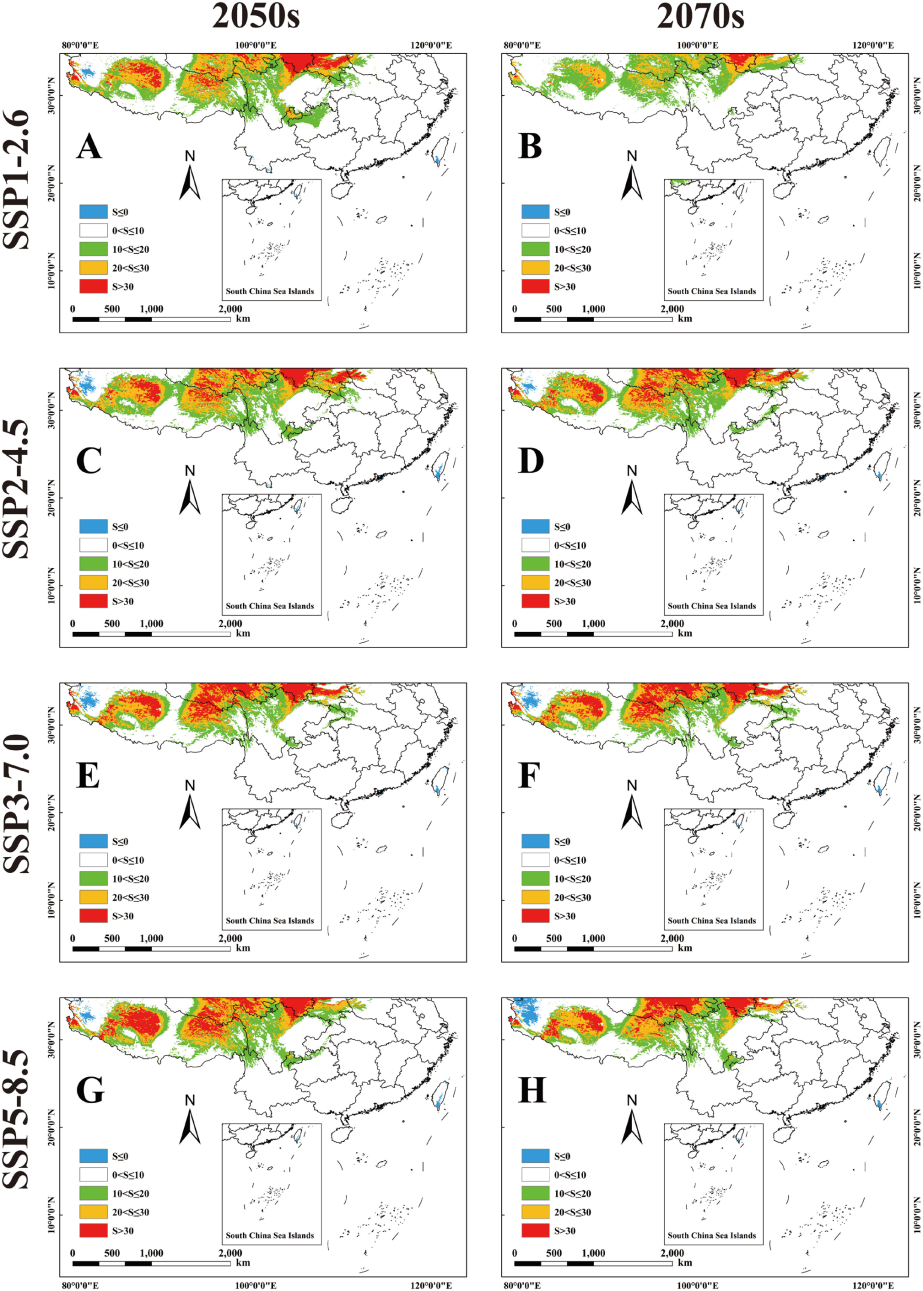
The multivariate environmental similarity surfaces (MESS) of *Pseudoechthistatus* in China under the future BCC‐CSM2‐MR climate model. Note: (A) SSP1‐2.6‐2050s; (B) SSP1‐2.6‐2070s; (C) SSP2‐4.5‐2050s; (D) SSP2‐4.5‐2070s; (E) SSP3‐7.0‐2050s; (F) SSP3‐7.0‐2070s; (G) SSP5‐8.5‐2050s; (H) SSPSSP5‐8.5‐2070s.

**FIGURE 7:**
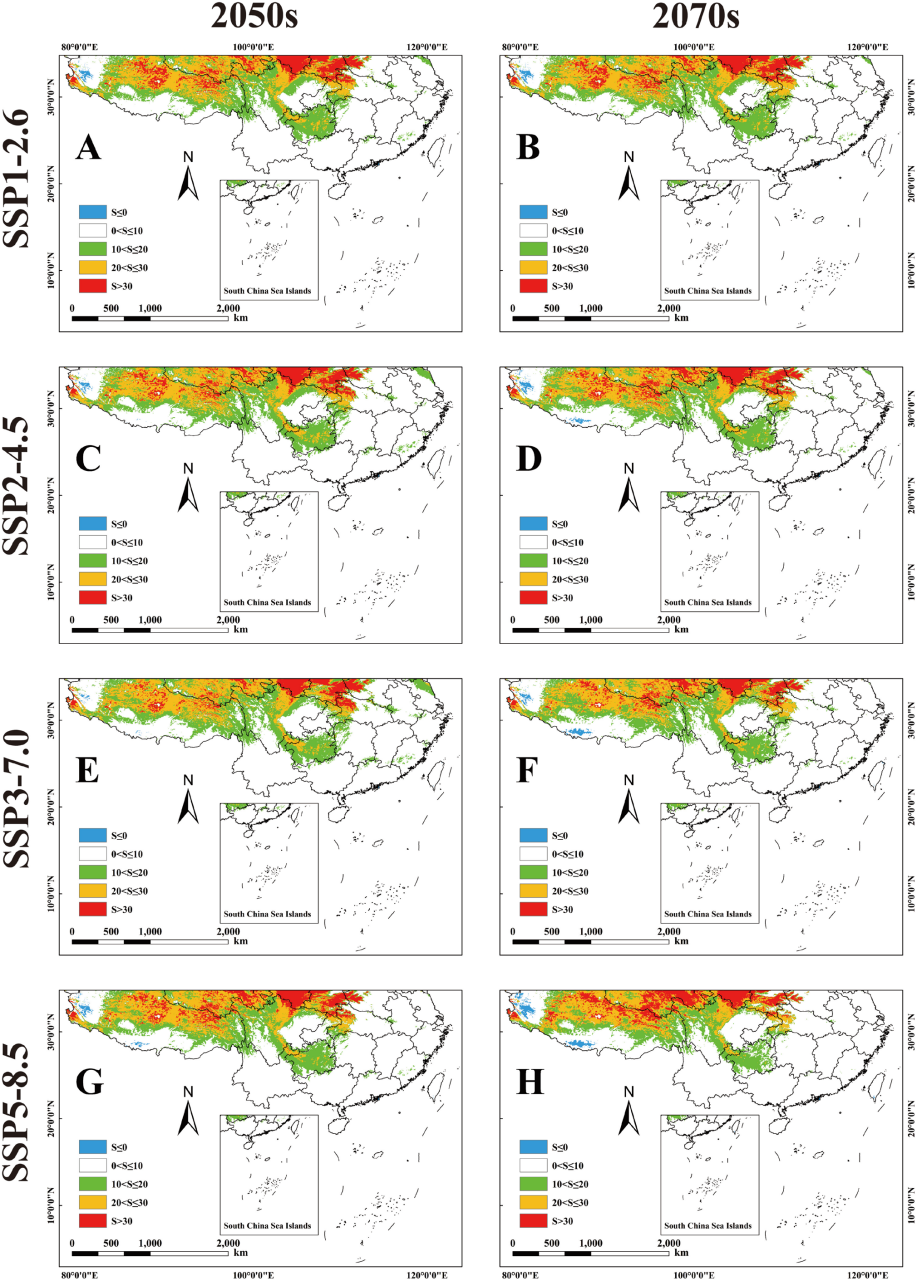
The multivariate environmental similarity surfaces (MESS) of *Pseudoechthistatus* in China under the future MIROC6 climate model. Note: (A) SSP1‐2.6‐2050s; (B) SSP1‐2.6‐2070s; (C) SSP2‐4.5‐2050s; (D) SSP2‐4.5‐2070s; (E) SSP3‐7.0‐2050s; (F) SSP3‐7.0‐2070s; (G) SSP5‐8.5‐2050s; (H) SSPSSP5‐8.5‐2070s.

The results show that the area of climate anomalies (S ≤ 0) in the potential geographic distribution of *Pseudoechthistatus* will expand by the 2050s and 2070s under four different scenarios and is time‐dependent, such that the area of S value increases with time, with the largest increase in S value under the SSP5‐8.5 scenario in the 2070s of the BCC climate model (Figure 6; Figure 7; Table S2). In addition, the provinces of Gansu, Qinghai, Shaanxi, Shanxi, and Ningxia, which are suitable for the distribution of *Pseudoechthistatus* under the future climate scenarios, have a high similarity to the climatic conditions in their places of origin (Yunnan and Sichuan) (Figure 7). The western part of the Tibetan Plateau is the least similar to the habitat of *Pseudoechthistatus*.

## 4 Discussion

Global climate change is altering the habitat patterns of many organisms, affecting species range, biodiversity, and ecosystem stability (Zhao et al. 2020). The genus *Pseudoechthistatus* is an important insect in forest ecosystems, and its distribution is strongly constrained by bioclimatic factors such as temperature and precipitation. However, there are still few studies on the habitat suitability and future distribution pattern of this genus. As an important tool for biodiversity research, ENMs can accurately simulate the potential distribution of species and assess their responses to environmental changes, providing a scientific basis for ecological conservation and management (Lwin et al. 2021; Zhang et al. 2024e). The MaxEnt model predicts the distribution of samples from the *Diamesus* Hope habitat with a high degree of reliability and an accuracy of 99.5% (Růžička et al. 2023). Similarly, the BIOCLIM model predicts that the potential high suitability area for *Sitobion* (Hemiptera: Aphididae) will increase from 41.3% to 53.3% in the future (Wang, Hof, et al. 2023). When compared with these models, MaxEnt can use the AUC value of the ROC curve to judge the model's predictions (Zheng et al. 2023). In this study, we applied the optimized MaxEnt model to predict the potential suitable distribution areas of *Pseudoechthistatus* under current and future climate scenarios and analyzed the effects of key environmental factors. The results not only revealed the spatial distribution pattern of suitable habitats for *Pseudoechthistatus* but also provided an important basis for evaluating the impact of climate change on its ecological dynamics, which is helpful for the development of scientific conservation strategies for the species.

This study shows that temperature and precipitation are the dominant environmental factors that determine the geographic distribution of *Pseudoechthistatus*. Among them, isothermality, temperature seasonality, and precipitation of driest quarter, with a cumulative contribution of 75.90%, were the key variables influencing the distribution of *Pseudoechthistatus* suitability. This result suggests that the species prefers to be distributed in regions with a more stable climate, less interannual fluctuations in temperature, and is more sensitive to extreme drought conditions. Higher isothermality and lower temperature seasonality may provide *Pseudoechthistatus* with more stable temperature environments, which in turn reduces physiological stresses due to drastic temperature fluctuations during development. Whereas in areas with high temperature fluctuations, insects may face higher energy expenditure to adapt to rapidly changing environments, stable temperature conditions may favor their growth and development and population reproductive capacity (Zhang et al. 2024d). Moreover, isothermality and precipitation of driest quarter indirectly affect the population dynamics of host plants by regulating their water status and food availability. In regions with large temperature fluctuations, *Pseudoechthistatus* may need to expend additional energy to adapt to extreme temperature changes, for example, by altering activity patterns, lengthening stasis periods, or adjusting metabolic levels in response to unfavorable temperature conditions. These adaptive strategies may reduce their survival and thus affect their geographic range. In addition, areas with higher seasonal temperatures tend to have more pronounced seasonal climatic variations, which may affect the growth cycle and health of host plants, and consequently the habitat stability of *Pseudoechthistatus* (Zhang et al. 2024e). Therefore, the habitat extent and population dynamics of *Pseudoechthistatus* may change significantly in the context of global warming and changing precipitation patterns. These findings not only provide a scientific basis for predicting the potential dispersal trend of *Pseudoechthistatus*, but also provide an important reference for biodiversity conservation and management strategies.

Under the current climatic conditions, the highly suitable areas for *Pseudoechthistatus* are mainly located in the Yunnan‐Guizhou Plateau, the eastern Himalayan Hengduan Mountains, and some mountainous areas, indicating that the species within the genus share similar aggregation characteristics in geographic distribution and prefer warm, humid montane forest ecosystems with small seasonal temperature variations. This prediction was highly consistent with actual occurrence records, further validating the reliability of the model. However, identifying potentially suitable habitats alone is not sufficient to fully assess the sustainability of species habitats. Future studies can incorporate landscape ecology models (e.g., Invest and Fragstats) to further analyze the degree of habitat fragmentation in potentially suitable areas and assess the impacts of anthropogenic disturbances on *Pseudoechthistatus* habitats to more accurately assess its habitat integrity, thus providing a scientific basis for protected area planning and habitat restoration (Meza Mori et al. 2023). This will not only help maximize the survival and reproduction of the species, but also promote the conservation and sustainable use of biodiversity, thereby maintaining the stability and integrity of the ecosystem (Zhou et al. 2023).

In the context of global warming, most species will face the threat of gradual habitat reduction or even loss, which is partly considered to be a scenario in which the future climate will lead to extinction and disappearance of species, with a reduction in global biodiversity (Mazziotta et al. 2022). In this study, we projected the trends of potential suitable habitats for *Pseudoechthistatus* in the 2050s and 2070s under different shared socio‐economic pathways scenarios based on two global climate models (BCC‐CSM2‐MR and MIROC6). The results indicated that under the BCC‐CSM2‐MR climate model, the suitable habitats of the genus showed a significant contraction trend, especially the area of highly suitable habitats was significantly reduced. It is important to note that while most studies show that the future climate will disrupt habitats and ecosystems, negatively affecting the survival and reproduction of species, some organisms will experience positive effects under future climate conditions (Sun et al. 2020; Kumar et al. 2022). The predictions of the MIROC6 model in this study indicated that the area of suitable habitat was expanding. This difference suggests that there is significant uncertainty in the predictions of future climate change among GCMs, leading to differences in the spatial pattern of suitable habitat for the species. The reason for the difference in the suitable area of *Pseudoechthistatus* may be related to the different simulation results of the two climate models on the future temperature and precipitation patterns, as BCC‐CSM2‐MR predicts that the future temperature increase will be larger, accompanied by stronger seasonal changes in precipitation, and that the extreme high temperatures and seasonal changes in temperature may exceed the physiological tolerance range of *Pseudoechthistatus*, resulting in the expansion of its suitable habitat. Under the BCC‐CSM2‐MR model, which predicts more temperature increases in the future, accompanied by stronger seasonal variations in precipitation, the extreme high temperatures and seasonal variations in temperature may exceed the physiological tolerance range of *Pseudoechthistatus*, leading to fragmentation of suitable habitat and thus further limiting its suitable distribution. In contrast, the MIROC6 model predicts a relatively mild future climate with less temperature variability, which may provide more stable moisture conditions for the forest ecosystems inhabited by *Pseudoechthistatus*, which is conducive to habitat maintenance and expansion. In addition, *Pseudoechthistatus* may be tolerant to changes in temperature and humidity, and thus the relative stability of suitable habitat under the MIROC6 model may be more consistent with its ecological needs. However, if the BCC‐CSM2‐MR projections of future climate are closer to reality, the risk of future habitat contraction for *Pseudoechthistatus* should not be ignored, especially under high emission scenarios, where the species may face more severe survival challenges. Therefore, in order to reduce the potential threat of climate change to this species, habitat protection and ecological restoration measures need to be strengthened in the future to enhance its adaptive capacity to environmental changes.

Driven by global climate change, the geographic distribution of many species is undergoing dynamic changes, often characterized by migration to higher latitudes or altitudes to adapt to changing environmental conditions (Niknaddaf et al. 2023). In the future, the suitable areas of *Pseudoechthistatus* show a tendency to migrate to higher latitudes and altitudes in search of more suitable habitats. This migration trend is consistent with the typical pattern of climate change—warmer climates prompt species to migrate to colder high latitudes and elevations in search of suitable temperature zones and resources. Although climate change is providing new habitat for *Pseudoechthistatus*, ecosystems at high latitudes and altitudes still face many challenges. Climate change not only affects species distributions through temperature increases, but is also accompanied by changes in precipitation patterns and an increase in extreme weather events, factors that may limit *Pseudoechthistatus*' adaptability to new environments, which in turn affects its ability to grow and reproduce during migration. Moreover, the survival of *Pseudoechthistatus* is not only directly affected by climatic conditions, but also constrained by factors such as host plant distribution and ecosystem structure. If the host plants fail to adapt to the new climatic conditions in a timely and synchronized manner, *Pseudoechthistatus* may face the risk of scarcity of food sources, which affects its survival and reproduction ability. In addition, the MESS analysis further verified the migration trend of *Pseudoechthistatus*' suitability zone. The results showed that under the two future climate models, the climatic conditions in northern China were more similar to those in the current period, which provided potentially suitable habitats for the migration and colonization of *Pseudoechthistatus*. This result suggests that climate change may lead to the spatial redistribution of species' suitable areas and prompt the migration of species to higher latitudes and altitudes. Thus, climate change not only affects the distribution areas of species, but may also drive ecological adaptation and growth dynamics of species in new areas, resulting in the emergence of new ecological patterns.

In species distribution simulations, the emergence of new ranges may be limited by the species' ability to migrate on its own, or by other dominant species occupying ecological niches in the area of migration, resulting in the failure of *Pseudoechthistatus* to form stable populations in these areas. At the same time, the migration of the species may be limited by geographic barriers or climatic adaptations that diminish its ability to expand into these areas. In addition, due to incomplete specimen records, certain habitats that are actually present may not have been detected or documented, resulting in actual distribution areas that may be smaller than potential distribution areas (Spaak et al. 2021; Hayat, Kour, et al. 2023). Future studies should enhance data collection and incorporate more environmental variables (e.g., vegetation type, topography, and anthropogenic factors) to further validate and refine the potential distribution predictions of *Pseudoechthistatus*. Therefore, timely management and conservation measures are essential to expand the range of *Pseudoechthistatus*, enhance species richness, and conserve biodiversity (Chen et al. 2022; Wedegärtner et al. 2022). Ecological restoration should be prioritized in potentially suitable habitats for *Pseudoechthistatus* to enhance habitat quality and ensure that the species can migrate and colonize new areas. In addition, the government and relevant organizations should strengthen the protection of critical habitats, reduce the disturbance of the ecological environment caused by human activities, and provide suitable ecological conditions for the species to migrate, so as to ensure the long‐term survival and reproduction of the species.

## 5 Conclusion

In this study, the optimized MaxEnt model was applied for the first time to predict and analyze the spatial distribution pattern and potential suitable areas for *Pseudoechthistatus* in China. The results show that *Pseudoechthistatus* prefers the temperate plateau monsoon climate, and suitable temperature and humidity conditions are more favorable for its growth, development, and migration. Under the current and future climate, the areas with higher suitability are mainly distributed in the vicinity of the Yunnan–Guizhou Plateau and the Himalayan Mountains, and the potential geographic distribution center of *Pseudoechthistatus* migrates towards the northwest. Additionally, in the future, focusing on areas with less temperature change is important for the continued survival of *Pseudoechthistatus* under the influence of climate change. This study elucidates the effects of climatic and environmental variables on the distribution of *Pseudoechthistatus* from a new perspective. The results help predict the direction of occurrence and migration patterns of *Pseudoechthistatus* under future climatic conditions and provide valuable guidance for taxonomists and researchers in searching for new species and refining the species diversity of the genus.

## Author Contributions

**Liang Zhang:** conceptualization (equal), methodology (equal), software (equal), writing – original draft (equal), writing – review and editing (equal). **Ping Wang:** funding acquisition (equal), investigation (equal), supervision (equal), writing – review and editing (equal). **Guang‐Lin Xie:** investigation (equal), supervision (equal), writing – review and editing (equal). **Wen‐Kai Wang:** funding acquisition (equal), supervision (equal), writing – review and editing (equal).

## Conflicts of Interest

The authors declare no conflicts of interest.

## Supporting information


Data S1.

## Data Availability

The authors confirm that the data supporting the findings of this study are available within the article and its Supporting Information S1.
