# Supplementary material for: Assessing the Potential Distribution of Pseudoechthistatus (Coleoptera: Cerambycidae) in China Under Climate Change Using Species Distribution Models
Source: Ecol Evol. 2025 Apr 14;15(4):e71303. doi: 10.1002/ece3.71303 (PMC11995185; doi:10.1002/ece3.71303)
Supplement: Supplementary file 1 — Data S1. [file ECE3-15-e71303-s001.docx]

**Evaluation of Potential Suitable Distribution Areas for Genus-Level Insects with Low Species Counts under Climate Change: A Case Study of *Pseudoechthistatus* Pic (Coleoptera: Cerambycidae) in China**

**Supplementary data**

**Table S1**

The occurrence records of *Pseudoechthistatus* in China.

| **Species** | **Longitude** | **Latitude** |
| --- | --- | --- |
| *Pseudoechthistatus obliquefasciatus* Pic, 1917 | 99.22166667 | 27.31833333 |
|  | 99.28188 | 27.34904 |
|  | 99.27661 | 27.34647 |
|  | 99.46305556 | 27.1925 |
|  | 101.2416667 | 25.15722222 |
| *Pseudoechthistatus sinicus* Bi & Lin, 2016 | 101.3766667 | 25.84444444 |
|  | 100.3972222 | 25.94277778 |
|  | 100.5736111 | 25.84055556 |
|  | 100.21 | 25.1 |
|  | 100.3919444 | 25.17722222 |
|  | 99.715 | 25.36305556 |
| *Pseudoechthistatus chiangshunani* Bi & Lin, 2016 | 101.2666667 | 24.32305556 |
|  | 100.8430556 | 24.44361111 |
|  | 101.2294444 | 24.19666667 |
|  | 99.56916667 | 24.23194444 |
|  | 100.4702778 | 24.59111111 |
| *Pseudoechthistatus holzschuhi* Bi & Lin, 2016 | 103.2833333 | 22.855 |
|  | 102.9933333 | 22.97555556 |
|  | 100.8330556 | 24.45055556 |
|  | 103.6972222 | 22.91 |
| *Pseudoechthistatus pufujiae* Bi & Lin, 2016 | 98.71388889 | 25.95638889 |
|  | 98.98944444 | 25.49888889 |
|  | 98.80666667 | 25.28944444 |
| *Pseudoechthistatus glabripennis* Bi & Lin, 2016 | 101.2486111 | 21.93555556 |
|  | 103.6968056 | 22.90641667 |
|  | 103.115424 | 22.551172 |
|  | 103.6869444 | 22.92138889 |
| *Pseudoechthistatus granulatus* Breuning, 1942 | 98.71021 | 27.1838 |
|  | 98.66583333 | 27.74972222 |
|  | 98.72694444 | 27.695 |
| *Pseudoechthistatus hei* Xie & W. Wang, 2019 | 99.17 | 27.09555556 |
| *Pseudoechthistatus rugosus* Huang, 2020 | 98.76833333 | 27.03888889 |
| *Pseudoechthistatus acutipennis* Chiang, 1981 | 103.3466667 | 29.55777778 |

**Table S2**

Multivariate environmental similarity surfaces (MESS) affecting the distribution of *Pseudoechthistatus* under future climate conditions.

| **General circulation model** | **Decade** | **Scenarios** | **S value area(10^4^ km^2^)** | | | | |
| --- | --- | --- | --- | --- | --- | --- | --- |
|  |  |  | **S ≤ 0** | **0 < S ≤ 10** | **10 < S ≤ 20** | **20 < S ≤ 30** | **S ≥ 30** |
| BCC-CSM2-MR | 2050s | SSP1-2.6 | 5.579861 | 535.6458 | 179.3108 | 115.2865 | 126.0122 |
|  |  | SSP2-4.5 | 4.213542 | 555.757 | 174.5469 | 102.9306 | 124.3872 |
|  |  | SSP3-7.0 | 2.248264 | 574.8351 | 152.8229 | 101.0174 | 130.9115 |
|  |  | SSP5-8.5 | 4.175347 | 600.408 | 140.0104 | 84.31424 | 132.9271 |
|  | 2070s | SSP1-2.6 | 6.253472 | 568.3386 | 165.2188 | 101.2708 | 120.7535 |
|  |  | SSP2-4.5 | 4.0625 | 574.4549 | 154.276 | 95.36111 | 133.6806 |
|  |  | SSP3-7.0 | 5.529514 | 604.9774 | 134.592 | 79.10069 | 137.6354 |
|  |  | SSP5-8.5 | 14.28472 | 643.5608 | 119.2951 | 73.17882 | 111.5156 |
| MIROC6 | 2050s | SSP1-2.6 | 1.751736 | 473.0226 | 184.9149 | 135.2361 | 166.9097 |
|  |  | SSP2-4.5 | 1.574653 | 474.8403 | 185.8819 | 137.0747 | 162.4635 |
|  |  | SSP3-7.0 | 0.954861 | 452.2587 | 202.0347 | 138.1111 | 168.4757 |
|  |  | SSP5-8.5 | 2.508681 | 480.8785 | 176.4097 | 133.3819 | 168.6563 |
|  | 2070s | SSP1-2.6 | 1.921875 | 479.3038 | 182.9115 | 134.2205 | 163.4774 |
|  |  | SSP2-4.5 | 3.043403 | 485.3142 | 176.4028 | 130.3333 | 166.7413 |
|  |  | SSP3-7.0 | 3.081597 | 487.1771 | 179.8194 | 130.5243 | 161.2326 |
|  |  | SSP5-8.5 | 6.78125 | 508.4774 | 166.8004 | 119.0781 | 160.6979 |


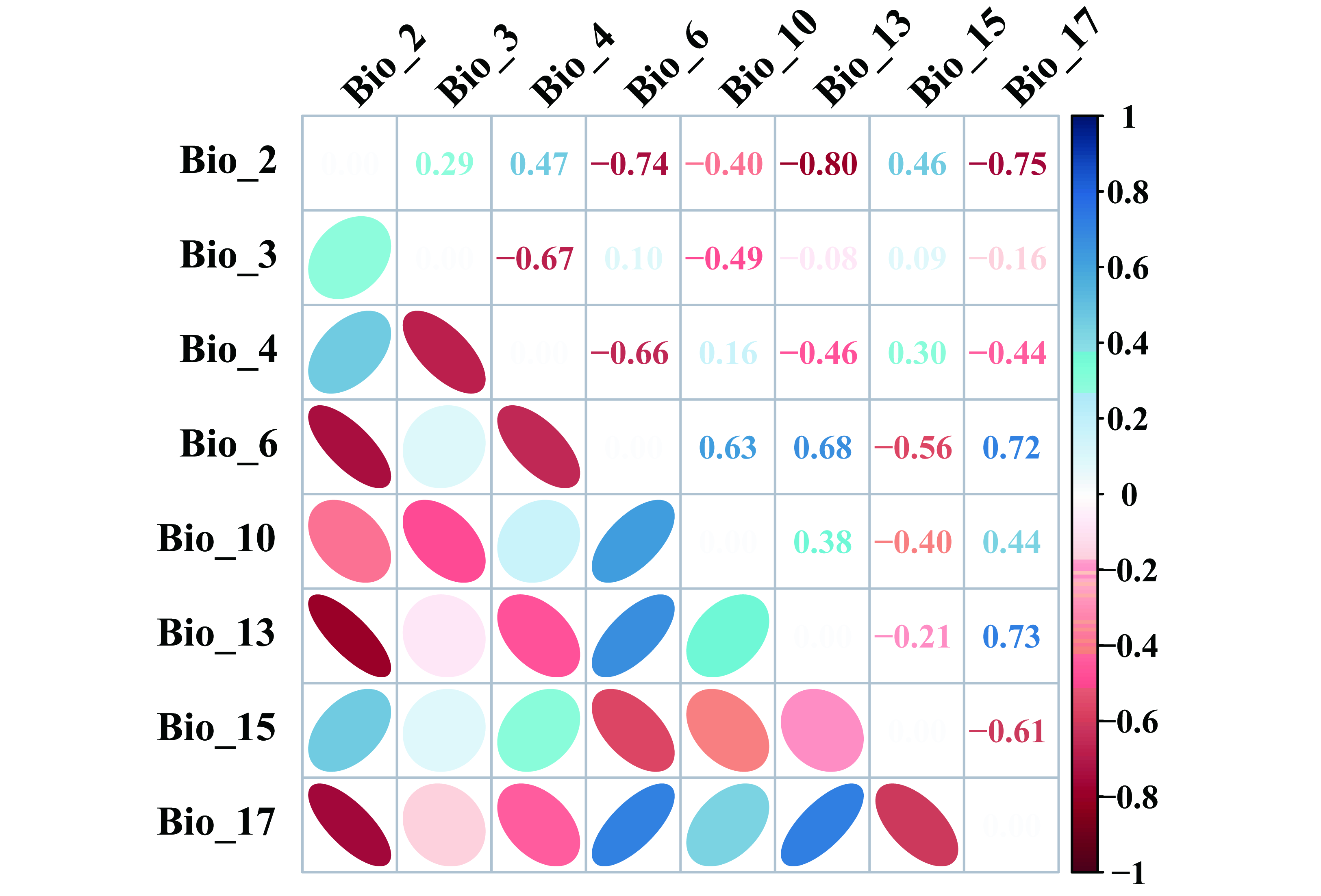


**Fig. S1** Correlation among the 8 bioclimatic variables.


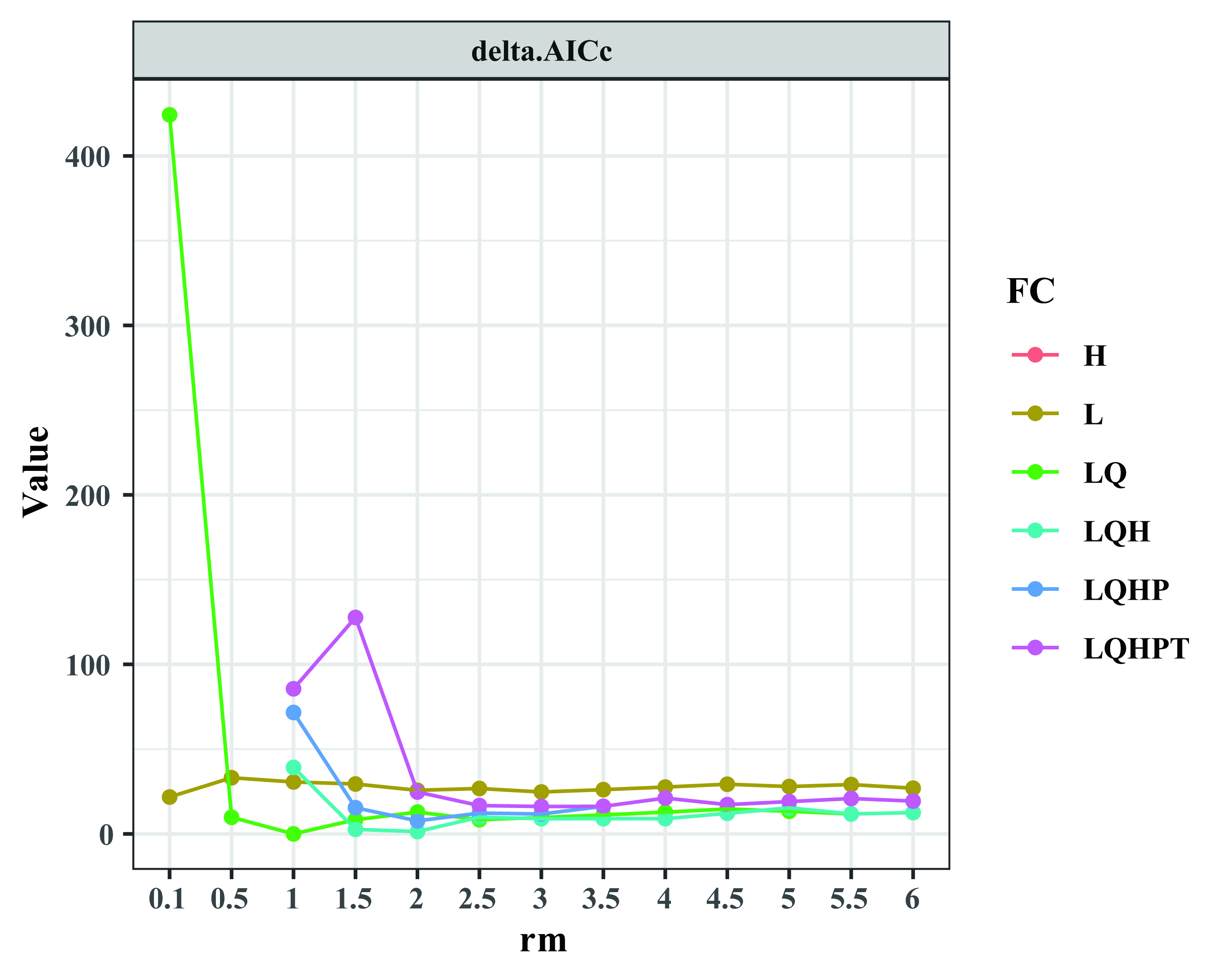


**Fig. S2.** Tuning parameters for *Pseudoechthistatus* in predicting potential distribution regions using the MaxEnt model.
